# CALENDAR

**DOI:** 10.1054/bjoc.2000.1352

**Published:** 2000-07-03


					3-8 September 2000
11th International Congress of Histochemistry and
Cytochemistry (ICHC 2000)
University of York, UK
Further information from: ICHC 2000 Secretariat, Royal
Microscopical Society, 37/38 St. Clements, Oxford OX4 1AJ,
UK. Tel: +44 (0) 1865 248768; Fax: +44 (0) 1865 791237; E-
mail: info@rms.org.uk; Website:
www.med.ic.ac.uk/external/ichc_2000/
28-30 September 2000
Seventh International Conference on Gastrointestinal
Oncology: Colorectal Cancer
Arcachon, France
Jointly sponsored by The George Washington University, Institut
Bergonie, and l'Universite Victor Segalen (University of
Bordeaux II).
Further information from:
Office of Continuing Medical Education, The George Washington
University Medical Center, 2300 K Street NW, Washington, DC
20037, USA. Tel: (202) 994 4285; Fax: (202) 994 1791.
11-13 December 2000
Genes & Cancer 2000
UK Molecular Biology & Cancer Network Meeting XVII
University of Warwick, UK
Transcriptional Control, Cytoplasmic Signalling, Nuclear
Structures, Cell Cycle Control, Therapeutics
Further information from:
Dr Stefan Roberts, Department of Biochemistry, University of
Dundee, Dundee, UK. Tel: + 44 (0) 1382 344248;
Fax: + 44 (0) 1382 345783; E-mail: s.g.e.roberts@dundee.ac.uk;
Website: www.icr.uk/ukmbcn/info.htm
7-10 March 2001
4th International Symposium on Leukemia and
Lymphoma - molecular pharmacology and new
treatment modalities
Amsterdam, The Netherlands
Chairman: G.J.L. Kaspers, R. Pieters, P. Sonneveld and A.J.P.
Veerman
Abstract deadline: 1 December 2000
Further information from:
VU Conference Service, De Boelelaan 1105, NL-1081 HV
Amsterdam, The Netherlands. Tel: + 31 (0) 20 444 5790;
Fax: + 31 (0) 20 444 5825; E-mail: vu_conference@dienst.vu.nl
CALENDAR
419
British Journal of Cancer (2000) 83(3), 419
(c) 2000 Cancer Research Campaign
doi: 10.1054/ bjoc.2000.1352, available online at http://www.idealibrary.com on

				

